# Avoidance and contextual learning induced by a kairomone, a pheromone and a common odorant in female CD1 mice

**DOI:** 10.3389/fnins.2015.00336

**Published:** 2015-10-06

**Authors:** Lluís Fortes-Marco, Enrique Lanuza, Fernando Martínez-García, Carmen Agustín-Pavón

**Affiliations:** ^1^Unitat Pre-departamental de Medicina, Facultad de Ciencias de la Salud, Universitat Jaume ICastelló de la Plana, Spain; ^2^Departament de Biologia Cel·lular, Facultat de Ciències Biològiques, Universitat de ValènciaValència, Spain

**Keywords:** aversion, isoamyl acetate, 2,4,5-trimethylthiazoline, 2-heptanone, kairomones, pheromones, place conditioning, vomeronasal

## Abstract

Chemosignals mediate both intra- and inter-specific communication in most mammals. Pheromones elicit stereotyped reactions in conspecifics, whereas kairomones provoke a reaction in an allospecific animal. For instance, predator kairomones elicit anticipated defensive responses in preys. The aim of this work was to test the behavioral responses of female mice to two chemosignals: 2-heptanone (2-HP), a putative alarm pheromone, and 2,4,5-trimethylthiazoline (TMT), a fox-derived putative kairomone, widely used to investigate fear and anxiety in rodents. The banana-like odorant isoamyl acetate (IA), unlikely to act as a chemosignal, served as a control odorant. We first presented increasing amounts of these odorants in consecutive days, in a test box in which mice could explore or avoid them. Female mice avoided the highest amounts of all three compounds, with TMT and IA eliciting avoidance at lower amounts (3.8 pmol and 0.35 μmol, respectively) than 2-HP (35 μmol). All three compounds induced minimal effects in global locomotion and immobility in this set up. Further, mice detected 3.5 pmol of TMT and IA in a habituation–dishabituation test, so avoidance of IA started well beyond the detection threshold. Finally, both TMT and IA, but not 2-HP, induced conditioned place avoidance and increased immobility in the neutral compartment during a contextual memory test. These data suggest that intense odors can induce contextual learning irrespective of their putative biological significance. Our results support that synthetic predator-related compounds (like TMT) or other intense odorants are useful to investigate the neurobiological basis of emotional behaviors in rodents. Since intense odorants unlikely to act as chemosignals can elicit similar behavioral reactions than chemosignals, we stress the importance of using behavioral measures in combination with other physiological (e.g., hormonal levels) or neural measures (e.g., immediate early gene expression) to establish the ethological significance of odorants.

## Introduction

Rodents are widely used in studies on the neurobiological basis of emotional behaviors. Chemical signals are the most relevant sensory cues for rodents, capable of eliciting strong emotional responses in them. For example, chemical signals such as alarm pheromones and predator kairomones are anxiogenic for mice and rats. Alarm pheromones are substances released by an injured or threatened animal and detected by conspecifics (Gutiérrez-García et al., 2007; Brechbühl et al., 2013), whereas predator kairomones elicit defensive responses in preys (for a review, see Fortes-Marco et al., 2013). Thus, experiments exposing mice and rats to alarm pheromones and kairomones are valuable to investigate the neural circuits controlling fear and anxiety, key features of pathologies such as generalized anxiety disorder, depression, or post-traumatic stress disorder.

The volatile chemical 2-heptanone (2-HP) has been proposed to act as an alarm substance in bees (Collins et al., 1989) and rats (Gutiérrez-García et al., 2007). This ketone is a component of the urine of rats, and its concentration is higher in stressed individuals. Urine from stressed rats or 2-HP alone induce stress-like reactions in recipient subjects (Gutiérrez-García et al., 2006), and despair in rats subjected to the forced swim paradigm (Gutiérrez-García et al., 2007). In mice, the concentration of 2-HP in urine is dependent on the adrenal gland (Novotny et al., 1986), and it acts as a puberty modulator in females in a blend with other chemicals (Novotny et al., 1986; Jemiolo et al., 1989). Thus, 2-HP might be classified as a trigger pheromone for rats and a primer pheromone for mice, following the definition by McClintock (2002). To our knowledge, the behavioral responses of mice to 2-HP have not been characterized yet, and most of the studies using 2-HP have focused on the mechanism of detection (Boschat et al., 2002; Thompson et al., 2004). Interestingly, a recent paper suggests that, in mice, some substances proposed as alarm pheromones, like 2-sec-butyl-4,5-dihydrothiazole, share structural similarity with kairomones (but see Jemiolo et al., 1985 on the possible role of this chemical as a sexual pheromone). Thus, alarm pheromones and kairomones might act using the same neural circuits conveying signals of danger (Brechbühl et al., 2013).

Cat fur and feces are potential sources of kairomones for rodents. Indeed, cat odors consistently induce biochemical and behavioral measures of stress in mice and rats, such as elevation of plasma glucocorticoids, fear responses such as freezing, avoidance, and contextual memory (Berton et al., 1998; Muñoz-Abellán et al., 2008, 2009). However, the components of cat fur odor and feces are diverse, not yet fully characterized, and the reaction to cat odor depends on the donor cat (Muñoz-Abellán et al., 2008) and its diet (Berton et al., 1998; Ferrero et al., 2011). Conversely, specific odorants derived from predator sources would offer the advantage of a controllable presentation to produce reproducible results.

The synthetic molecule 2,5-dihydro-2,4,5-trimethylthiazoline (TMT), a component isolated from fox feces (Vernet-Maury, 1980), offers the mentioned advantages. TMT induces freezing, contextual learning, and anxiety-related behavioral changes in rodents (Endres and Fendt, 2007, 2009). Moreover, TMT activates brain regions related to stress and anxiety, although the activation pattern differs from that induced by cat odor (Dielenberg et al., 2001; Staples et al., 2008; Janitzky et al., 2015; Pérez-Gómez et al., 2015). This fact, together with the fact that both unconditioned responses to TMT and its ability to support conditioning are dependent on the environment and protocol used (Wallace and Rosen, 2000; Morrow et al., 2002; Blanchard et al., 2003; Endres and Fendt, 2007), have led to some debate on the kairomonal or simply aversive nature of this molecule (Fendt and Endres, 2008; Fortes-Marco et al., 2013).

In this vein, we have previously hypothesized that the exposure to intense odorants might be indeed very aversive, so it could elicit similar anxiety-like responses to the ones elicited by kairomones and alarm pheromones in mice—not necessarily accompanied by behavioral components of fear i.e., freezing (Fortes-Marco et al., 2013).

Thus, we sought to compare the behavioral reactions of female mice to increasing amounts of TMT, 2-HP and isoamyl acetate (IA), a banana-like odorant frequently used as a control odor (Wallace and Rosen, 2000; Root et al., 2014) in two-choice tests. Previous studies from our lab have validated these tests to investigate the attractive properties of male pheromones for female mice (Agustín-Pavón et al., 2007, 2014; Martínez-Ricós et al., 2007). We hypothesized that the putative kairomone TMT would be avoided at detectable concentrations. The putative pheromone 2-HP might be either attractive or aversive depending on its role—alarm pheromone or puberty modulator, see above. Finally, the control odorant should not elicit avoidance, except perhaps at high concentrations. We also hypothesized that sufficiently aversive olfactory stimulation might be able to support learning in the animals. Thus, we tested whether repeated exposure to the concentrations eliciting the maximum behavioral reaction could induce a conditioned contextual avoidance in mice.

## Materials and methods

### Animals

For the present experiments, we used 125 adult female CD1 mice (8–18 weeks old, Janvier SAS, St Berthevin Cedex, France). Animals were housed under controlled temperature (22–24°C) and a light-dark cycle 12:12 (light from 08:00 to 20:00), with food and water *ad libitum*. Animals were treated throughout according to the European Communities Council Directive of (86/609/EEC), and the protocols were approved by the Committee of Ethics on Animal Experimentation of the University of Valencia.

### Odorants

We used three odorants: 2,5-dihydro-2,4,5-trimethylthiazoline (TMT; Contech, Victoria, Canada), a putative kairomone found in fox feces and detected by the main olfactory system and the Grueneberg ganglion (Brechbühl et al., 2013), 2-heptanone (2-HP, Sigma-Aldrich, Schnelldorf, Germany), a putative mouse pheromone detected by the main and accessory olfactory epithelia (Thompson et al., 2004; Xu et al., 2005), and isoamyl acetate (IA, Panreac Quimica SA, Barcelona, Spain), a control odorant detected by the main olfactory system (Xu et al., 2005) and unlikely to participate in intra or interspecific communication (Root et al., 2014). We diluted the odorants to the desired concentration, with PBS with Triton X-100 1%, (pH = 7.4) for 2-HP and TMT and mineral oil for IA. We selected these solvents because the odorants were more readily diluted in them than in distilled water. In addition, mice were not able to detect differences between the odorants diluted in the solvents or in distilled water (Supplementary Material, Experiment S1). Also, the solvents did not elicit enhanced chemoinvestigation (see Section Results and **Figure 2**).

### Behavioral tests

#### Experiment 1. behavioral effects of increasing amount of odorants

To check the behavioral reaction of mice toward each amount of odorant in two-choice tests, subjects were randomly assigned to four groups. Each group was presented with one of the odorants or PBS (control) (TMT *n* = 12, 2-HP *n* = 11, IA *n* = 12, PBS *n* = 12). The behavioral test was performed in a methacrylate opaque box (45 × 47.5 × 22.5 cm), divided in two identical chambers by a panel with a door to minimize diffusion of the odors. Each chamber had one perforated stainless steel capsule (JP Selecta, Abrera, Barcelona, Spain) attached to the floor with double-sided adhesive tape. We prepared serial dilutions of each of the odorants, from the pure substance to 10^−8^, and pipetted 5 μl of the corresponding solution in a piece of filter paper (2 × 2 cm) inside each capsule. The capsule on the stimulus chamber contained 5 μl of the corresponding odorant, whereas the capsule on the neutral chamber contained the corresponding solvent, except in the PBS group, in which both capsules contained 5 μl of PBS.

Animals were habituated to the experimenter and apparatus for 10 min for 3 days. The fourth day, we performed a 5-min test (control), in which both capsules contained the correspondent solvent (PBS with Triton X-100 or mineral oil, see above) or PBS. On the following days, mice were exposed to the 5 μL of the relevant chemical stimulus at increasing concentrations at the stimulus chamber (exposure days 1–9), and 5 μL of the corresponding solvent in the capsule at the neutral chamber. All odorants have similar molecular weight and density (TMT 129.2, 1 g/ml; 2-HP 114.2, 0.8 g/ml; IA 130.2, 0.9 g/ml), so the molar concentration of the pure substance is similar (TMT 7.7 M, 2-HP and IA, 7 M). Thus, the first exposure day, mice had access to 0.38 pmol of TMT or 0.35 pmol of 2-HP and IA, and the amount of odorant was increased 10-fold each day to the pure substance, i.e., 38 μmol of TMT or 35 μmol of 2-HP and IA. To avoid diffusion of the odorant and facilitate exhausting the volatiles of the room, we performed the tests in a room with mild negative air pressure. Experimental cages were thoroughly cleaned after each test, and each stimulus was used in a different room.

#### Experiment 2. habituation–dishabituation tests

To establish the detection threshold of each of the odorants, we submitted three separate groups of mice (*n* = 6 for each odorant) to habituation–dishabituation tests, following Agustín-Pavón et al. (2007).

The tests were performed in a squared opaque methacrylate box (25 cm^2^) with a hole at 8 cm from the floor in one of the walls. Females were placed in the test box 3 min before the test for habituation. The stimuli were presented to the mice in a stick with a cotton swab at the tip, which was introduced through the hole and fixed to the box wall. We presented the mice with three consecutive 1-min presentations of the swab impregnated with 5 μl of water, followed by three consecutive presentations of 5 μl each odorant amount. Between each odorant amount, mice were presented with three consecutive 1-min presentations of the corresponding solvent. Mice investigate more each stick when presented for the first time or when its odor changes, and investigation decreases in successive presentations. Thus, this is a reliable method to investigate the detection threshold of odorants.

#### Experiment 3. place conditioning test

To test whether repeated exposure to the odorants in the same location would induce learning, we performed a place conditioning test following the protocol described in Martínez-Ricós et al. (2007). Animals were randomly distributed in three groups, (TMT, *n* = 11; 2-HP, *n* = 12, IA, *n* = 12). We used the same test box as in Experiment 1, so that animals could freely explore both chambers. Animals were habituated to the experimental conditions for 10 min for 2 days. The third day, we put 5 μL PBS in each capsule and recorded the behavior of the animals for 5 min (control). From the next day, mice had access for four consecutive days (training days, 1–4) to 5 μL of their corresponding odorant at pure concentration in the stimulus chamber and to PBS in the neutral chamber, for 5 min. The day after the last training session, we evaluated the induction of contextual memory (test) with PBS in each capsule.

### Behavioral measures

All tests were video-recorded and the videos were analyzed with the video-tracking software SMART v2.5.11 (Panlab, Cornella, Spain). For experiments 1 and 3, we defined an area of interest covering 25% of each chamber surface, as a circular region of 12 cm of radius surrounding the center of the capsule (stimulus or neutral zones). This zone ensured the detection of the animal in close proximity of the stimuli.

For each stimulus and test, we obtained data from time spent in the area of interest and distance traveled in cm. Informal observations by a trained observer who was blind to the experimental conditions revealed lack of risk assessment postures or freezing, so we used the percentage of time that the animal moved at a speed < 1 mm/s as an approximate measure of immobility (Fortes-Marco et al., 2013). As a measure of attraction/avoidance, we calculated an avoidance ratio as the ratio between the time spent on the stimulus zone and the total time spent in the neutral plus the stimulus zone (see Agustín-Pavón et al., 2014). A value of 0.5 of this avoidance ratio indicates that the stimulus is neither attractive nor avoided, whereas an avoidance ratio < 0.5 reveals avoidance of the stimuli. For experiment 2, the tests were videotaped and an observer blind to the experimental conditions measured the time that females spent rearing on their hind limbs and actively sniffing at the cotton tip.

### Statistical analysis

Data were analyzed with R statistical software (v. 3.1.2, http://www.R-project.org/) and IBM SPSS 22.0. We checked the normality and homocedasticity of the data by means of a Kolmogorov–Smirnov and Levene test. Data from Experiment 1 were analyzed by means of ANOVA for repeated measures, with the factors DAY/CONCENTRATION (for the avoidance ratio) and ZONE (for time spent in the zones) as within-subject factors, followed by Dunnet *post-hoc* comparisons (to compare exposure days with the control condition) or *post-hoc* pairwise comparisons with the Bonferroni correction. Data from Experiment 2 were analyzed by means of paired Student's *t*-tests between the last presentation of each solvent and the first presentation of the following odorant. Data from Experiment 3 were analyzed by means of paired Student's *t*-test (avoidance ratio in control vs. place avoidance test), repeated measures ANOVA (distance traveled and immobility) with DAY and ZONE as within-subjects factors, and repeated measures ANOVA (avoidance ratio during exposure days) with DAY as within-subject factor and ODOR as between-subjects factor.

## Results

### Experiment 1. behavioral effects of increasing amount of odorants

The aim of the first experiment was to determine the range of amounts at which each odor would elicit a measurable behavioral reaction in mice, including attraction/avoidance, distance traveled, and immobility, in two-choice tests. To ensure that there was no *a priori* preference of the animals for any compartment—this was unlikely, since both were identical-, or some habituation process that would affect the activity of the animals across tests, we run group of animals that were exposed to PBS in each zone for 10 consecutive days. None of the behavioral measures varied across days in these mice exposed to PBS alone, so any behavioral changes in the mice exposed to the different odorants could be attributed to the effects of the stimuli. (Supplementary Material, Experiment S2).

**Figure 1:**
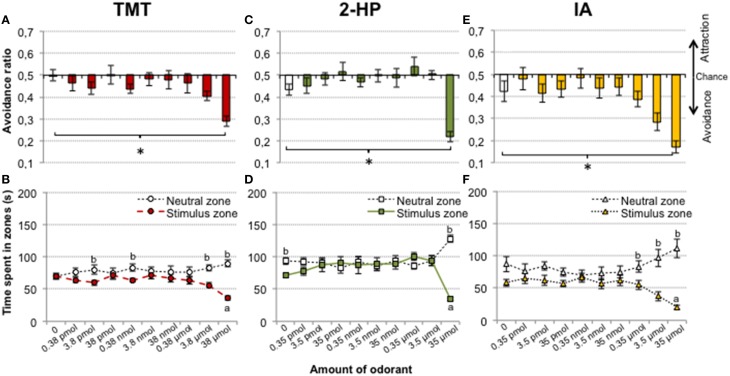
**TMT, 2-HP, and IA induced avoidance in a concentration-dependent way**. Bar and line charts representing the avoidance ratio and time spent in each zone for increasing concentrations of each odorant. For all three odorants, the avoidance ratio was significantly different from the control situation (no odor) only when pure substance was presented (**A,C,E**, ^*^p < 0.05). Time spent by the animals at the stimulus zone was dose-dependently decreased by TMT with respect to the neutral zone **(B)**, whereas 2-HP did not induce avoidance except when presented pure **(D)**. Finally, IA produced a pattern of avoidance similar to that of TMT **(F)**. Comparison between the stimulus zone with the same zone in the control (no odor): a, *p* < 0.05. Comparison between the stimulus and the neutral zones: b, *p* < 0.05. Data are represented as mean ± SEM.

The avoidance ratio for the group exposed to TMT was significantly different from control at the highest amount of TMT used [repeated measures ANOVA, factor CONCENTRATION, *F*_(9, 99)_ = 4.9, *p* < 0.001; *post-hoc* comparison between exposure day 9 (38 μmol of TMT) vs. control (no TMT), *p* = 0.009; Figure 1A]. We further checked whether there was a difference in the raw time that animals spent in each zone. There was a significant decrease of the time spent in the stimulus zone when it contained 38 μmol of TMT with respect to the stimulus zone in the control day [repeated measures ANOVA, CONCENTRATION × ZONE, *F*_(9, 99)_ = 3.6, *p* < 0.001; *post-hoc* comparison for exposure day 9, stimulus zone vs. control, *p* = 0.001; Figure 1B]. Still, *post-hoc* pairwise comparisons between zones showed that time spent in the TMT zone was lower with respect to the neutral zone during exposure days 2, 4, 8, and 9 (3.8 pmol, *p* = 0.023; 0.38 nmol, *p* = 0.015; 3.8 μmol, *p* = 0.002; and 38 μmol, *p* < 0.001; Figure 1B). These results suggest that mice were able to detect TMT from 3.8 pmol, since this amount induced a slight avoidance reaction (Figure 1B), but TMT was strongly avoided at pure concentration only as measured with both time spent in zones and avoidance ratio.

The putative pheromone 2-HP induced avoidance at the highest amount presented [repeated measures ANOVA of the avoidance ratio, CONCENTRATION, *F*_(9, 90)_ = 9.4, *p* < 0.001, *post-hoc* comparison exposure day 9 vs. control, *p* = 0.001; Figure 1C]. Further, the ANOVA of the raw time spent in zones and subsequent *post-hoc* tests revealed an increase in time spent in the neutral zone and a decrease in the stimulus zone with 35 μmol of 2-HP [CONCENTRATION × ZONE, *F*_(9, 90)_ = 7.4, *p* < 0.001, *post-hoc* exposure day 9 vs. control, neutral zone *p* = 0.009, stimulus zone *p* = 0.03; Figure 1D]. Pairwise *post-hoc* comparisons between zones confirmed a significant difference of time spent in stimulus vs. neutral zone in exposure day 9 only (*p* < 0.001).

Finally, mice also avoided IA at the highest amount presented [repeated measures ANOVA for the avoidance ratio, CONCENTRATION, *F*_(9, 90)_ = 9.1, *p* < 0.001; *post-hoc* comparison of exposure day 9 vs. control, *p* = 0.048; Figure 1E]. For the time spent in each zone, there was a decrease in time spent in the stimulus zone in the presence of 35 μmol of IA with respect to the control [ANOVA, CONCENTRATION × ZONE, *F*_(9, 90)_ = 5.83, *p* < 0.001, *post-hoc* comparison of exposure day 9 vs. control, *p* = 0.002]. In addition, *post-hoc* pairwise comparisons revealed significantly lower time spent in the stimulus zone as compared to the neutral zone when IA was presented from 0.35 to 35 μmol (*p* = 0.014; *p* = 0.004; *p* < 0.001, respectively; Figure 1F). Thus, the pattern of avoidance of IA resembles that of TMT.

Surprisingly, an ANOVA between tests revealed that TMT did not significantly affect distance traveled [*F*_(9, 90)_ = 1.7, *p* = 0.11] or percentage of immobility [*F*_(9, 90)_ =1.5, *p* = 0.22]. By contrast, distance traveled was, overall, significantly decreased by the increasing concentrations of 2-HP [DAY effect, *F*_(9, 90)_ = 4.0, *p* < 0.001]. *Post-hoc* comparisons revealed significant decreases in the presence of 0.35 nmol to 3.5 μmol of 2-HP with respect to the control (all *p* < 0.05). The percentage of immobility was also significantly different across tests [DAY, *F*_(9, 90)_ = 2.2, *p* = 0.027]. Thus, 2-HP was avoided by mice at the highest presented amount, but concentration-dependently affected the activity of the animals. Finally, the exposure to IA significantly decreased the distance traveled and increased immobility globally across tests [*F*_(9, 90)_ = 3.4, *p* = 0.018, *F*_(9, 90)_ = 3.3, *p* = 0.026; Table 1].

**Table 1:** **Exposure to the TMT did not significantly affect distance traveled or immobility, whereas both 2-HP and IA decreased distance traveled and increased percentage of immobility across tests**.

**Odorant**		**Day 1**	**Day 2**	**Day 3**	**Day 4**	**Day 5**	**Day 6**	**Day 7**	**Day 8**	**Day 9**	**Day 10**
		**0**	**0.35–0.38 pmol**	**3.5–3.8 pmol**	**35–38 pmol**	**0.35–0.38 nmol**	**3.5–3.8 nmol**	**35–38 nmol**	**0.35–0.38 μmol**	**3.5–3.8 μmol**	**35–38 μmol**
TMT	Distance (cm)	2080 ± 156	1995 ± 143	1914 ± 124	1881 ± 118	1781 ± 83	1817 ± 97	1944 ± 111	1883 ± 85	1895 ± 73	1919 ± 80
	Percentage of immobility	13.7 ± 1.3	14.8 ± 1.4	15.2 ± 1.3	16.1 ± 1.4	16.7 ± 1.0	16.3 ± 1.0	14.9 ± 1.1	15.1 ± 1.0	15.3 ± 0.9	14.2 ± 0.9
2-HP	Distance (cm)	2380 ± 114	2278 ± 103	2156 ± 124	2147 ± 84	2000 ± 60	1939 ± 50	1946 ± 51	2133 ± 76	1914 ± 78	2143 ± 73
	Percentage of immobility	11.6 ± 0.9	12.6 ± 1.1	14.1 ± 1.1	13.3 ± 0.7	14.2 ± 0.9	14.5 ± 0.8	14.8 ± 0.9	13.5 ± 0.8	15.0 ± 0.8	12.7 ± 0.8
IA	Distance (cm)	2024 ± 109	1836 ± 109	1853 ± 97	1919 ± 140	1786 ± 111	1752 ± 101	1725 ± 106	1812 ± 111	1762 ± 131	1639 ± 118
	Percentage of immobility	17.8 ± 1.3	20.7 ± 1.8	19.9 ± 1.8	19.6 ± 1.8	20.9 ± 1.6	20.9 ± 1.5	22.3 ± 1.6	21.0 ± 1.8	21.2 ± 2.0	23.9 ± 2.7

### Experiment 2. habituation–dishabituation test

**Figure 2:**
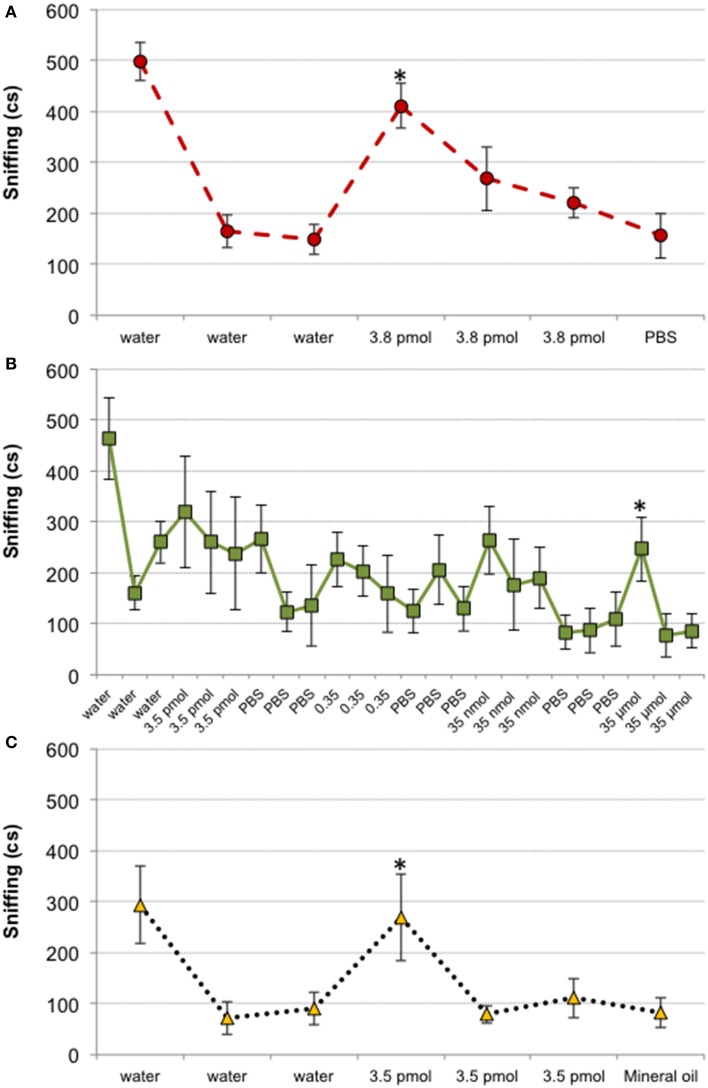
**Mice are able to detect TMT, 2-HP and IA in habituation–dishabituation tests**. Line charts representing the chemoinvestigation of scented cotton swabs in cs. 3.8 pmol of TMT elicited increased chemoinvestigation **(A)**, whereas 2-HP did not significantly increase chemoinvestigation until we presented 35 μmol **(B)**. The detection threshold of IA was similar to that of TMT **(C)**. Comparison between last presentation of a solvent (water, PBS with Triton X-100 1% or mineral oil) and first presentation of each odorant amount, ^*^p < 0.05. Data are expressed as mean ± SEM.

Results from Experiment 1 suggested that mice could detect 3.8 pmol of TMT, since this was the minimal amount that elicited a slight avoidance response. However, both 2-HP and IA were not avoided until we presented 35 and 0.35 μmol, respectively. To investigate whether odorants affected the behavior of mice at their olfactory detection threshold or beyond, we carried out a habituation–dishabituation test. The analysis comparing the last presentation of the cotton swab impregnated with solvent with the first presentation of each amount of odorant tested revealed that female mice detected 3.8 pmol of TMT, since this amount increased investigation of the cotton swab (*p* = 0.002; Figure 2A). By contrast, 2-HP did not significantly increased investigation until 35 μmol were presented (*p* = 0.039; Figure 2B). Finally, 3.5 pmol of IA significantly increased chemoinvestigation (*p* = 0.005). Thus, we confirmed that mice detected 3.8 pmol of TMT, although avoidance behavior was not strong with this amount of odorant. Further, in agreement with the avoidance expressed by mice in Experiment 1, 2-HP did not significantly enhanced chemoinvestigation until presented at the maximum amount (see also Supplementary Material). By contrast, IA was detected in the habituation–dishabituation test much more diluted than it was avoided in Experiment 1. These results also raise the possibility that learning could contribute to the strong avoidance displayed toward TMT and IA at the highest amount used. Finally, this experiment confirmed that the solvents did not elicit chemoinvestigation (Figure 2).

### Experiment 3. place conditioning test

We next checked whether the repeated exposure to the different stimuli was able to induce the formation of a contextual memory. To do so, we used the undiluted odorants, which were equally avoided by mice, in a place conditioning experiment.

**Figure 3:**
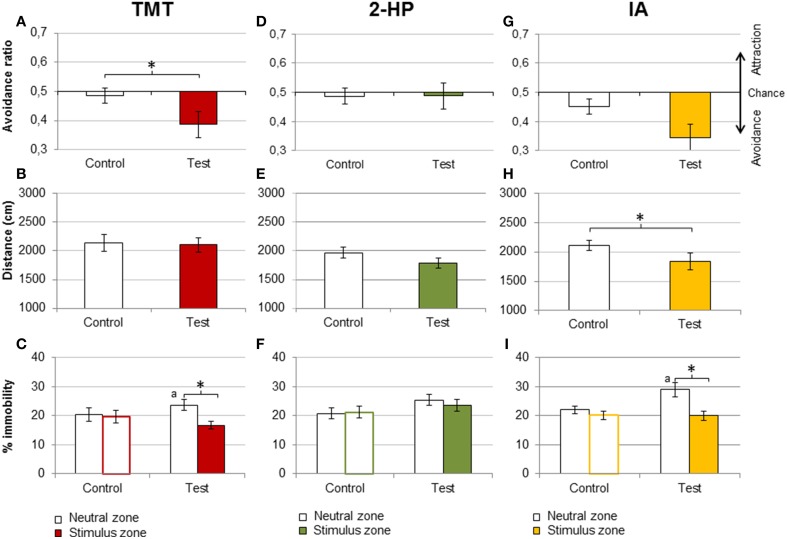
**TMT and IA, but not 2-HP, induced contextual learning**. Bar charts representing behavioral responses in the contextual learning experiment, in the pre-exposure control (Control) and the contextual memory test (Test). Mice exposed to pure TMT during four consecutive sessions expressed conditioned place avoidance to the zone paired with the stimulus, as shown by a significant decrease of the avoidance ratio in the test **(A)**. In addition, TMT failed to affect distance traveled **(B)**, but increased immobility in the neutral zone during the test **(C)**, suggesting that animals avoided TMT and stayed immobile in the neutral zone. By contrast, 2-HP did not induce a contextual memory, since it did not affect the avoidance ratio **(D)**, but, overall, it slightly decreased distance traveled **(E)**, and did not affect immobility **(F)**. Finally, IA produced a marginally significant decrease in the avoidance ratio **(G)**, and significantly decreased distance traveled **(H)**. Like TMT, IA increased immobility in the neutral zone **(I)**. ^*^p < 0.05. Comparison between the neutral zones in control and test: a, *p* < 0.05. Data are expressed as mean ± SEM.

Mice of the TMT group expressed a conditioned avoidance for the stimulus chamber after four consecutive days of exposure to this kairomone (Student's *t*-test of the avoidance ratio control vs. place conditioning test, *p* = 0.029; Figure 3A). Moreover, a Student's *t*-test against the chance value 0.5 revealed that the avoidance ratio was significantly different from chance in the test (*p* = 0.027) but not the pre-training control (*p* = 0.64). However, neither distance traveled nor global percentage of immobility were significantly different between the control and the place conditioning test (*p* = 0.7, Figure 3B). We further explored whether the percentage of immobility would be dependent on the zone, i.e., whether mice would stay inactive in the neutral or the stimulus zone (Fortes-Marco et al., 2013). Indeed, there was a significant difference between zones in the place conditioning test, driven by an increase in the percentage of immobility in the neutral zone in the test with respect to control [repeated measures ANOVA, DAY × ZONE, *F*_(1, 10)_ = 8.7, *p* = 0.014, *post-hoc* stimulus vs. neutral zone in the test, *p* = 0.002; *post-hoc* time spent in neutral zone in control vs. test, *p* = 0.017; Figure 3C].

In spite of being strongly avoided at the highest amount presented, 2-HP did not induce contextual memory (avoidance ratio, control vs. test *p* = 0.99; Figure 3D). Distance traveled and immobility were not significantly affected either (*p* = 0.068 and 0.094, respectively; Figures 3E,F).

Finally, in the group exposed to IA the decrease of the avoidance ratio did not reach statistical significance with respect to the control (*p* = 0.067; Figure 3G). However, a Student's *t*-test against the chance value revealed that the avoidance ratio was significantly different from chance at the test (*p* = 0.001) but not the control (*p* = 0.115), so this measure indicates the formation of a conditioned avoidance. Further, distance traveled was significantly lower in the test with respect to the control (*p* = 0.034; Figure 3H). The percentage of immobility in each zone was different in the memory test, due to an increase in immobility in the neutral zone [DAY × ZONE, *F*_(1, 11)_ = 4.9 *p* = 0.049, stimulus vs. neutral chamber, *p* = 0.004; time in immobility in the neutral zone control vs. test, *p* = 0.012; Figure 3I).

In summary, TMT and IA induced a contextual memory after repeated exposure, so that mice avoided the stimulus zone even in the absence of the odorants. Exposure to IA significantly decreased distance traveled in the contextual memory test, and both TMT and IA increased the immobility of the animals in the neutral zone, paralleling the significant avoidance of the stimulus zone. Conversely, exposure to 2-HP did not produce statistically significant behavioral differences between the control and the memory test.

**Figure 4:**
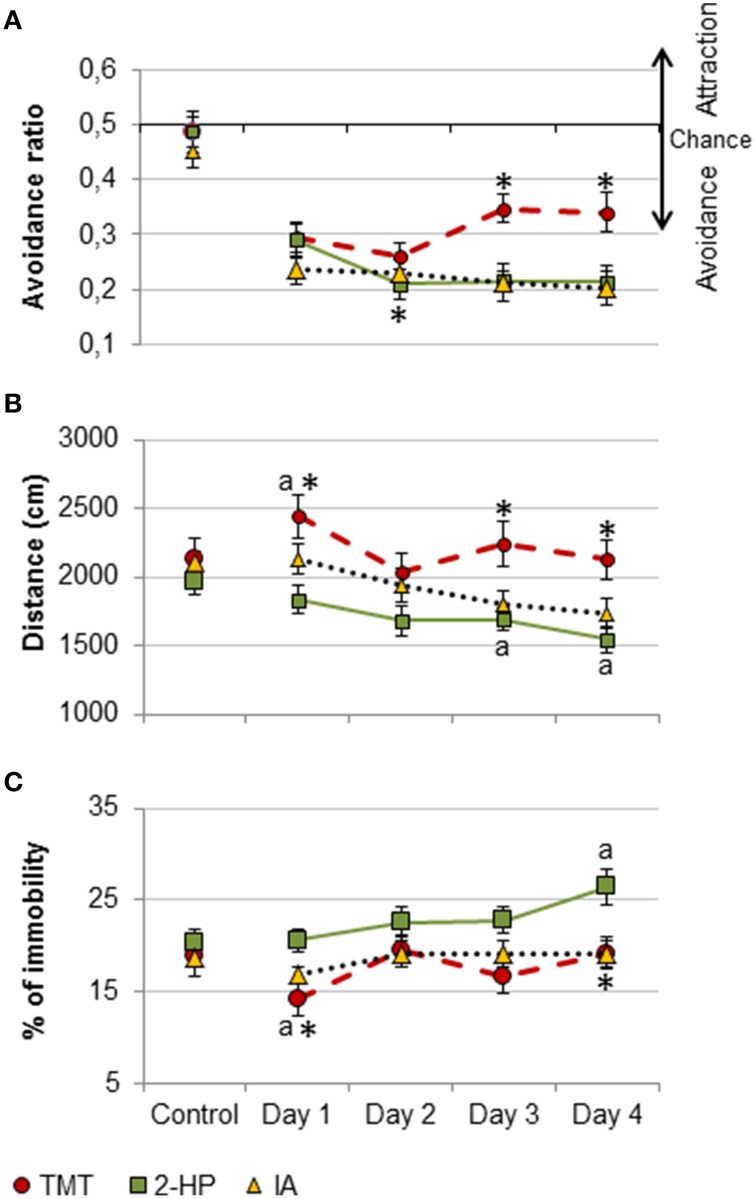
**Pure TMT, 2-HP and IA were avoided and differently affected locomotion**. Line charts representing behavioral responses during the training days of the contextual conditioning experiment for each odorant. All three odors were significantly avoided, but the avoidance of TMT was blunted across sessions, whereas avoidance of 2-HP increased in the second day **(A)**. Distance traveled was differentially affected by the three odors: whereas TMT increased locomotion during the first exposure day and then this parameter remained stable, 2-HP and IA decreased locomotion across tests **(B)**. TMT reduced immobility the first training day, and 2-HP increased it during the last training day, whereas IA did not affect to this measure **(C)**. Comparison between odorants: ^*^p < 0.05. Comparison between exposure days and the control situation: a, *p* < 0.05. Data are expressed as mean ± SEM.

To check whether the different effects of each odorant in memory and activity were due to differential effects of the repeated exposure to them, we compared the avoidance ratio, distance traveled and immobility during the training days between groups (TMT, 2-HP and IA). The avoidance ratio of the groups exposed to TMT and 2-HP was significantly different to those of IA [repeated measures ANOVA, DAY × ODOR, *F*_(6, 96)_ = 3.1, *p* = 0.01; *post-hoc* comparison TMT vs. IA and 2-HP and 2-HP vs. IA, both *p* = 0.023; Figure 4A]. Further *post-hoc* pairwise comparisons between the individual exposure days revealed that all odors were equally avoided during the first two training days, but from day 3, the avoidance ratio of TMT decreased and became significantly different from both 2-HP and IA (*p* < 0.05 in all cases; Figure 4A). In fact, the avoidance ratio in the TMT group was significantly different between day 2 and 3 (*p* = 0.042). Conversely, the avoidance ratio of the group exposed to 2-HP was significantly lower in day 2 than in day 1 (*p* = 0.02). Finally, the avoidance ratio of the group exposed to IA did not vary across days. These findings suggest that mice were slightly habituated to the aversive properties of TMT, whereas they expressed a higher avoidance of 2-HP in consecutive tests.

Distance traveled was significantly different across exposure days and between groups [repeated measures ANOVA, ODOR, *F*_(2, 32)_ = 5.4, *p* = 0.009; TEST × ODOR, *F*_(6, 96)_= 3.1, *p* = 0.01; Figure 4B]. *Post-hoc* comparisons of the factor ODOR showed that, overall, the distance traveled by the mice exposed to TMT was significantly different to distance traveled by the mice exposed to 2-HP (*p* = 0.007). Further *post-hoc* pairwise comparisons revealed that distance traveled by animals exposed to 2-HP was lower at day 4 as compared to day 1 (*p* = 0.014), whereas mice exposed to IA traveled less distance all three last exposure days as compared to the first one (all *p* < 0.05). We thus checked whether these differences were due to basal differences in distance traveled between the groups. Importantly, distance traveled was similar for all groups at the control day (*p* > 0.1). Further, the ANOVA followed by Dunnet *post-hoc* comparison revealed that TMT increased distance traveled during the first exposure with respect to the control (*p* = 0.019), whereas 2-HP and IA decreased distance traveled with respect to the control at days 3 and 4, respectively (both *p* = 0.04).

As expected, immobility followed a pattern that was complementary to that of distance traveled [repeated measures ANOVA, TEST × ODOR, *F*_(6, 96)_ = 2.3, *p* = 0.042]. Overall, immobility was significantly different in the TMT and 2-HP groups (*p* = 0.037). Again, these differences were not due to different initial levels of immobility, since this measure was not significantly different between groups in the control day (all *p* > 0.9). In contrast to most reports showing an increase in freezing in animals exposed to TMT, this putative kairomone decreased immobility during the first day as compared to control (*p* = 0.017). Conversely, 2-HP increased immobility at day 4 as compared to control (*p* = 0.004), whereas animals exposed to IA did not show any significant variation in the percentage of time they spent inactive (Figure 4C).

## Discussion

Our results indicate that female mice displayed specific behavioral changes when exposed to substances thought to be implicated in inter (TMT) and intraspecific communication (2-HP), but also to a common odorant unlikely to act as a chemosignal (IA). Mice avoided these chemicals in a concentration-dependent way, but showed opposite changes in activity in response to them. Further, avoidance of both TMT and IA was expressed beyond the detection threshold of the odorants. In addition, only TMT and IA induced conditioned, context-dependent behavioral changes. We discuss these results by comparing them with the behavioral responses of mice to the common odorant IA.

### Exposure to TMT elicits avoidance but not freezing

Mice mainly rely on their sense of smell to avoid danger. Thus, when given the opportunity, they readily avoid spots containing predator kairomones (Root et al., 2014; Wernecke et al., 2015). Our results in female CD1 mice, showing that they avoid a zone containing TMT, a putative kairomone, are in agreement with this view. In fact, in Experiment 1, mice spent less time in the zone of the cage containing TMT than in the opposite neutral zone when TMT was present at amounts as low as 3.8 pmol and 0.38 nmol (Figure 1A). However, in the ensuing sessions at higher concentrations (3.8 nmol to 0.38 μmol) mice seemed to habituate and spent the same amount of time in both zones. Avoidance reappeared at the highest concentrations (3.8 and 0.38 μmol). In Experiment 2, we confirmed the olfactory detection of 3.8 pmol TMT, so that mice can detect and avoid TMT at low concentrations, supporting its role as a kairomone.

However, the time spent in the stimulus zone was significantly reduced with respect to the control situation (no odor) only when 38 μmol TMT were presented (Figure 1B). In addition, mice displayed a similar avoidance pattern to TMT and to IA, an odorant unlikely to have a kairomonal role. In fact, IA is frequently used as a control odor for TMT studies (Wallace and Rosen, 2000; Root et al., 2014), and our results showing that low amounts of IA are detected but not avoided by mice support its use as such, albeit only at those low concentrations. Our findings suggest that the behavioral reactions that mice display to supra-threshold, very intense odorants might not be odorant-specific, and therefore they may not be informative about the role of that substance as a chemical signal. In this line, a recent study by Dewan et al. (2013), showed that genetic deletion of specific olfactory receptors blocked the aversion that mice expressed for low, but not high, concentration of amines found in the urine of predators and for the urine itself. The high concentration of amines used in that study were pungent to humans, so the aversion could be due to either overactivation of non-specific olfactory receptors or trigeminal stimulation (see below), supporting that that aversion toward very intense odors might not only depend on the biological significance of the odorant. Second, this study highlights that biologically significant chemosignals are detected at very low concentrations.

Further, behavioral avoidance of a given stimulus does not necessarily reflect fear, but rather that the stimulus has aversive properties for the subject due to its repugnant, pungent or disgusting properties. In this sense, Endres and Fendt (2009) demonstrated that whereas both TMT and butyric acid elicited avoidance in rats, only TMT elicited freezing, a more accurate behavioral measure of fear.

However, TMT did not produce any freezing in our mice as measured by a trained observer. Immobility, a behavioral measure that could approximate freezing, was not affected either by the increasing concentrations of TMT. Quite the opposite, animals repeatedly exposed to pure TMT displayed heightened locomotion and reduced immobility, as compared to both the control situation (no odor) and to animals exposed to 2-HP. These findings contrast with the above mentioned study by Endres and Fendt (2009) and several other reports showing that rats (Wallace and Rosen, 2000) and mice (Hebb et al., 2004; Hacquemand et al., 2013) display enhanced immobility and freezing in the presence of TMT, but are in agreement with our previous findings in two strains of mice (Fortes-Marco et al., 2013). In fact, sensitivity to TMT is dependent on the strain in rats, so that, under the same conditions, Sprague–Dawley, but not Wistar rats display freezing in the presence of TMT (Rosen et al., 2006). In agreement, we have previously shown that mice of the C57BL/J6 strain displayed enhanced immobility in the presence of the TMT as compared to CD1 (Fortes-Marco et al., 2013).

The contrasting findings about freezing to TMT found in the literature also reflect that responses of animals toward a given aversive stimulus are critically dependent on the protocol used. To investigate this issue, Morrow et al. (2002) exposed rats to TMT either in a low anxiety (a comfortable, dimly lit open field) or a high anxiety environment (a bigger apparatus intensely lit). Their results showed that rats displayed enhanced immobility in the high anxiety environment only. Thus, the behavioral strategy that animals select to cope with an aversive stimulus is dependent on the possibilities offered by the environment. In a single-chamber open field or in the home cage, where the animals cannot escape or hide, it is more likely that they freeze than in our set up, a box divided in two chambers with a neutral compartment where the animals can escape. This is also true in response to a footshock, the most common fear-inducing stimulus in the laboratory. When rats are given the opportunity to escape to a save chamber, they do not freeze but quickly escape following the footshock (Blanchard et al., 2006).

Additionally, the conditions in which volatile compounds are tested determine their effective concentration in the air. Thus, if a given amount of a volatile substance is presented in a small cage covered by a lid, the concentration in the air will be higher than in an open or larger cage. This might also explain the differences found between the different studies in the behavioral response of animals to volatiles.

### Exposure to pure 2-HP elicits avoidance and enhanced immobility

To our knowledge, this is the first report analyzing the behavioral effects of 2-HP in adult female mice. Although sometimes quoted as a “known mouse pheromone” (Xu et al., 2005), studies on the behavioral and endocrine effects of 2-HP in mice are scarce, and restricted to early reports about its puberty-affecting properties (Novotny et al., 1986; Jemiolo et al., 1989). Conversely, more recent studies in rats have shown that 2-HP is elevated in the urine of stressed rats (Gutiérrez-García et al., 2006) and increases immobility in the forced swimming test (Gutiérrez-García et al., 2007), suggesting that 2-HP acts as an alarm pheromone in this species (but see also Zhang et al., 2008; Zhang and Zhang, 2011 for studies about the role of 2-HP as a sexual pheromone in rats).

The alarming properties of 2-HP have been also demonstrated in bees (Collins et al., 1989). Even if it might seem surprising that the same compound acts as a pheromone in vertebrates and insects, this is not an isolated case. For example, (Z)-7-dodecen-l-yl acetate works as a pheromone in several species of moths and in elephants (Rasmussen et al., 1996). Anyway, although the avoidance observed to pure 2-HP in mice would fit its role as an alarm pheromone, it is possible that these aversive properties are due to pungent or disgusting properties of the pure substance—which the animals are unlikely to encounter in nature—rather than to its pheromonal actions. This might represent another case of substance-unspecific aversion to an intense odor (see above).

Although 2-HP elicited avoidance only when presented pure, it decreased the distance traveled and increased immobility in a concentration-dependent way. This finding suggests that 2-HP was detected at relatively low concentrations (0.35 nmol). However, data from habituation–dishabituation tests (Experiment 2 and Experiment S1) shows that amounts lower than 35 μmol of 2-HP did not significantly elicit chemoinvestigation. These results raise the possibility that 2-HP might have subthreshold behavioral effects. In fact, the vomeronasal organ of mice responds to 2-HP at a concentration of 10^−11^ *in vitro* (Leinders-Zufall et al., 2000). Moreover, this response is sex-specific, so that female-derived vomeronasal preparations, but not male-derived ones, responded to 2-HP by increasing the intracellular concentration of inositol-3-phosphate (Thompson et al., 2004), thus indicating that the aversion to the pure substance described in the present work might not be the only biological effect of this chemical signal. In summary, it is possible that the effects of 2-HP depend on the age and sex of the animals, so that it might act as a puberty regulator in pre-puber females, and as an alarm pheromone in adult females and males. The effects of 2-HP might also be strongly dependent on its concentration and on the chemical blend in which it is encountered (Novotny et al., 1986). Future studies are needed to tests these hypothesis.

Finally, the modulation of locomotor activity by IA and 2-HP was similar. Our data about the responses of mice to IA are in agreement to a previous study showing that IA induced avoidance and increased immobility in rats (Wallace and Rosen, 2000). Thus, although IA is commonly used as a control odor, and our data shows that it is neutral to mice at its detection threshold, our data also suggest that it should not be used at high concentrations for that purpose, since a strong olfactory stimulation results in strong avoidance.

In summary, the similarity in the behavioral responses of mice to high concentrations of two biologically significant chemosignals and a common odorant suggest that strong odorants might induce odorant-unspecific behavioral responses of avoidance. In mice, pure TMT elicits corticosterone secretion (see Fendt et al., 2005), and a pattern of brain c-fos expression suggestive of intense stress (Janitzky et al., 2015). These findings together with our present results suggest that intense odors might be used to study stress and anxiety in addition to other unspecific stimuli such as loud noises (Burow et al., 2005; Mikheenko et al., 2010). Studies of the endocrine response to and central effects of high concentrations of control odorants such as isoamyl acetate are needed to check this hypothesis.

### Conditioned contextual responses of mice after repeated exposure to TMT and IA

The lack of consistent freezing to TMT, together with the failure to induce the contextual learning that is observed after exposure to other predator cues, i.e., cat odor (Wallace and Rosen, 2000; Blanchard et al., 2003; Muñoz-Abellán et al., 2009) have been considered by several researchers as a challenge to the kairomonal nature of this substance. Nonetheless, Endres and Fendt (2007) explored whether TMT could support learning by using different conditioning protocols. Their data showed that whereas TMT failed to induce conditioning in rats trained in a box with a single compartment [in agreement with the results obtained by Blanchard et al. (2003)], it induced conditioned avoidance in a box divided in two compartments. Our results replicate and extend these findings, since mice in our experiment expressed a conditioned avoidance of a zone paired with TMT, as well as a specific increase in immobility in the neutral zone. This latter result is striking, since TMT decreased immobility during training days. Nevertheless, as noted by (Endres and Fendt, 2007), the conditioned behavioral responses are not necessarily the same as the unconditioned ones. In addition, it is possible that our mice were able to express conditioned avoidance because they could use TMT as a discriminative stimulus due to the lack of strong innate responses toward TMT, which could interfere with conditioning in other strains. Finally, as we have stressed before, differences in the set up and protocols used might contribute to differences in the experiments outcome.

On the contrary, 2-HP failed to induce a conditioned avoidance, albeit it was, overall, significantly more avoided than TMT during the exposure days. Moreover, whereas the avoidance toward 2-HP increased the second training day, the avoidance toward TMT was slightly reduced after repeated exposures, suggesting a slight habituation. These differences are open to several interpretations.

First, it is possible that, at the high concentration used, TMT activated the trigeminal nerve (Galliot et al., 2012) producing an aversion strong enough to form an avoidance memory. In fact, although trigeminal deafferentiation does not block freezing to TMT (Ayers et al., 2013), TMT induces trigeminal activation at concentrations higher than 10% (Hacquemand et al., 2010), at least in some experimental conditions. The exposure to IA, also known to stimulate the trigeminal nerve (Doty et al., 1978), mimicked the results obtained with TMT in our experiments. This suggests that a strong olfactory stimulation, maybe along with trigeminal activation, is enough to support learning, even if the odorant has, in principle, no special innate meaning for the animals. Future studies investigating memory induction with lower amounts of TMT and IA in control and anosmic animals would shed light on whether the olfactory and/or trigeminal properties of these odorants are responsible for their ability to support learning.

However, although 2-HP could also stimulate the trigeminal nerve at high concentrations (Cometto-Muñiz and Cain, 1995), and elicited aversion in mice when presented pure, it failed to induce contextual learning. Maybe 2-HP is recognized as a mouse-derived odor and, hence, it is not tagged as dangerous. Finally, whereas TMT and IA activate the main olfactory bulb only (Bepari et al., 2012), 2-HP is detected by both, the olfactory epithelium (Spehr et al., 2006) and V1R vomeronasal receptors (Boschat et al., 2002), thus activating both the main and the accessory olfactory systems (Xu et al., 2005). Given the overlapping but complementary roles of the main and accessory olfactory systems (Martínez-García et al., 2009), it is possible that higher order brain structures processing the olfactory and vomeronasal stimuli result in different unconditioned and learnt responses.

### Neural basis of biologically significant odor processing

The brain circuits processing TMT and other predator-derived cues (e.g., cat odor) might be, in fact, underlying the diversity of responses toward each type of chemosignals. Thus, cat fur odor activates the vomeronasal system (Dielenberg et al., 2001; Staples et al., 2008), including the posteroventral medial amygdala, a key center for defensive anti-predatory responses (Day et al., 2004; Pérez-Gómez et al., 2015). By contrast, TMT is not able to induce c-fos in this nucleus (Day et al., 2004; Janitzky et al., 2015; Pérez-Gómez et al., 2015). In agreement with the activation of defensive nuclei of the brain, mice show robust risk assessment responses toward cat odor (Pérez-Gómez et al., 2015), but fail to do so when exposed to TMT (Pérez-Gómez et al., 2015, present results). In this sense, cat fur odor seems a more valuable stimulus than TMT to study antipredatory responses in rodents.

On the other hand, the bed nucleus of the stria terminalis (BNST) seems key in controlling the behavioral responses to TMT. Thus, TMT elicits robust and specific increases of Fos induction in the BNST (Janitzky et al., 2015), and temporary inactivation of this nucleus with muscimol injections abolished freezing to TMT in rats (Fendt et al., 2003). The BNST has been related to anxiety rather than fear (Davis et al., 2010). Fear is a response to an explicit threat, whereas anxiety involves uncertainty as to the expectancy of threat, and predator-derived odors are, indeed, poor predictors of the presence of a predator itself (Blanchard et al., 2003). Thus, we suggest that TMT could certainly be regarded as an anxiogenic rather than as a fear-provoking stimulus, but this hypothesis needs further investigation.

Regarding 2-HP, to our knowledge there are not studies looking at the activation of central brain structures beyond the olfactory bulbs (Xu et al., 2005). Mapping of alarm pheromones obtained from the anal glands of rats revealed Fos increases in the BNST and other nuclei involved in stress processing (Kiyokawa et al., 2005). It would be interesting that future studies directly compare the brain activation patterns elicited by both biologically significant and common of odors under the same conditions.

## Conclusions

In conclusion, our results suggest that some intense odors are avoided by mice irrespective of their possible role in inter- or intra-species communication. Second, some of these intense odors, including fox-derived TMT and the odorant IA, which in principle is devoid of value for intra- or interspecies communication, induce conditioned avoidance when repeatedly presented at high concentration. By contrast, 2-HP is avoided at high concentrations but it does not induce contextual learning, maybe reflecting its role as an intraspecific chemosignal. These results support the widely use of synthetic predator-related compounds (like TMT) to investigate the neurobiological basis of emotional behaviors in rodents. However, since IA, a common odorant frequently used as control, elicits similar behavioral reactions to TMT, we stress the importance of using behavioral measures in combination with other physiological or neural manipulations (e.g., measurements of hormonal levels, expression of immediate early genes) to establish the ethological significance of chemosignals.

## Author contributions

EL, FM, and CA, designed research; LF and CA performed research; LF and CA analyzed data; LF, FM, and CA wrote the paper, EL, FM, and CA revised the final version and approved the manuscript.

### Conflict of interest statement

The authors declare that the research was conducted in the absence of any commercial or financial relationships that could be construed as a potential conflict of interest.
