# Supplementary material for: Avoidance and contextual learning induced by a kairomone, a pheromone and a common odorant in female CD1 mice
Source: Front Neurosci. 2015 Oct 6;9:336. doi: 10.3389/fnins.2015.00336 (PMC4594011; doi:10.3389/fnins.2015.00336)
Supplement: Supplementary file 1 [file DataSheet1.DOCX]

**Supplementary material**

*Experiment S1. Lack of influence of the solvent on the avoidance/detection of the odorant.* Mice had access to three consecutive one-minute presentations of the cotton swab impregnated with 5 μl of water, followed by three consecutive presentations of 3.5-3.8 μmol (10^-1^) of each odorant in 5 μl of either the solvent (PBS- 1% Tx100, mineral oil) or distilled water. The order of the solvent was counterbalanced, so three mice had access to the odorant dissolved in water first and three had access to the odorant dissolved in the corresponding solvent first. The first presentation of the odorant in either solvent elicited a peak in the average investigation, albeit this increase failed to be significant since some animals avoided investigating the stick, a result consistent with the avoidance seen in Experiment 1. (2 out of 6 avoided smelling TMT, 1 out of 6 avoided smelling both 2-HP and IA). TMT, p=0.36; 2-HP, p=0.07; IA, p=0.14. The change in the solvent did not increase or decrease the investigation, suggesting that this change did not affect the olfactory properties of the odorants.

*Experiment S2. Repeated exposure to the test box did not significantly affect to avoidance ratio, distance travelled or immobility.* A group of animals was exposed to PBS in each zone for 10 consecutive days. The statistical analysis confirmed that the avoidance ratio did not significantly vary across days (repeated measures ANOVA, Day 1 to 10, F_9,99_=1.2, p=0.32). Since none of the individual avoidance ratios were significantly different from the chance value, we concluded that there was no *a priori* preference or avoidance of either side of the cage (one sample Student’s t test against the probe value 0.5, all p>0.1). Thus, any deviations from chance in the avoidance ratio in the mice exposed to the different odorants could be attributed to avoidance/preference for the stimuli. In addition, we found no significant effects of repeated testing in either distance travelled or percentage of immobility (repeated measures ANOVA, all p>0.1), suggesting that repeated testing did not significantly alter the behavioral responses.

|  | **Day 1** | **Day 2** | **Day 3** | **Day 4** | **Day 5** | **Day 6** | **Day 7** | **Day 8** | **Day 9** | **Day 10** |
| --- | --- | --- | --- | --- | --- | --- | --- | --- | --- | --- |
| **Avoidance ratio** | 0.51±0.04 | 0.45±0.04 | 0.56±0.05 | 0.51±0.05 | 0.48±0.04 | 0.54±0.03 | 0.57±0.04 | 0.52±0.03 | 0.56±0.05 | 0.49±0.04 |
| **Distance (cm)** | 2481±265 | 2291±200 | 2388±254 | 2278±245 | 2362±246 | 2278±218 | 2206±208 | 2179±153 | 2206±142 | 2140±126 |
| **% of immobility** | 10.7±1.4 | 11.9±1.5 | 12.0±1.7 | 13.0±1.9 | 11.2±1.6 | 12.4±1.5 | 12.6±1.6 | 12.0±1.5 | 12.1±1.2 | 12.4±1.1 |

.
